# Is the meniscus posterior root the “Death Zone” of the knee joint?

**DOI:** 10.1002/jeo2.70277

**Published:** 2025-05-19

**Authors:** Angelo V. Vasiliadis, Vasileios Giovanoulis, Dimitrios Chytas, Luca Macchiarola

**Affiliations:** ^1^ Department of Orthopaedic Surgery St. Luke's Hospital Panorama Greece; ^2^ Department of Orthopaedic Surgery Croix‐Rousse Hospital Lyon France; ^3^ Orthopedic Department Centre Hospitalier de Versailles – Hopital Andre Mignot Le Chesnay Rocquencourt France; ^4^ Basic Sciences Laboratory, Department of Physiotherapy University of Peloponnese Sparta Greece; ^5^ Department of Orthopedics and Trauma Surgery Fondazione Casa Sollievo Della Sofferenza IRCCS San Giovanni Rotondo Italy

**Abbreviations**
mm: millimeters
m: meters

The meniscus has a well‐recognized crucial multifunctional role in the function of the knee joint, including load transmission, shock absorption, stability, lubrication, proprioception and nutrient supply [11]. Thus, more and more surgeons support the idea to ‘Save the meniscus’, underlying the importance of the meniscus integrity in the prevention of early degenerative alterations in the knee joint [10]. It is also supported that the meniscus preservation should be part of every surgeon's treatment algorithm with an increased need to aggressively repair every amenable meniscal pathology, such as ramp lesions, radial tears, bucket handle tears and root tears. Meniscal root tears are defined either as an avulsion of the meniscal insertion or a complete radial tear within 10 mm of the meniscal root insertion [1]. Interestingly, the recent recognition of pathology of meniscal root tears to alter joint biomechanics and accelerate articular cartilage degeneration, has characterized this type of meniscal injury as a ‘silent epidemic’ of the knee joint [2].

In mountaineering, the higher we go, the less oxygen we have in order to breathe, while passing an altitude above 8000 m, the body enters what climbers call the ‘Death Zone’. As surgeons, who deal with meniscal injuries, we would like to answer the question; is the meniscus posterior root the ‘Death Zone’ of the knee joint? Paraphrasing the ‘Death Zone’ of 8000 m in mountains, in the knee joint and especially in the meniscus, the more posterior we go, the more compressive forces and shear stresses are exposed to the meniscus, between the posterior femoral condyle and tibial plateau during deep flexion, increasing the risk of meniscal root tears. It is well documented in the literature that the natural history of an unrepaired meniscal posterior root tear can progressively lead to functional alterations, joint space narrowing and other degenerative changes in the knee joint, increasing the prevalence of a future arthroplasty procedure [2, 3].

It is noteworthy that recently published cadaveric, biomechanical and clinical studies have elucidated this pathology, underlying the necessity of meniscal posterior root repair [5, 6, 7, 8, 9]. From a biomechanical point of view, a meniscal posterior root tear and the loss of associated function is equivalent to a total meniscectomy, leading to an increase in pressure of the affected compartment and subsequently rapid development of osteoarthritis [9]. Lee et al., in their systematic review, tried to examine the radiological and clinical outcomes after repair, partial meniscectomy and nonoperative treatment in the management of meniscal root tears [6]. They found that meniscal posterior root repair may be the most viable treatment option in lessening joint space narrowing and producing improvements in patient‐reported outcomes, as measured by the International Knee Documentation Committee and Lysholm scores. Despite the preferred surgical repair technique, such as all‐inside repair, transtibial pullout repair and suture anchor repair, meniscal posterior root repair can effectively reduce the joint contact pressure and stress, while restoring the joint contact area to levels similar to those of the intact meniscus [5, 7, 8]. However, multiple factors, such as age, obesity, high posterior tibial slope, varus malalignment > 5°, meniscus midbody extrusion and tear gap, must be considered before the repair due to the high failure of healing after meniscus posterior root repair [4, 5, 8].

In summary, indeed, the meniscal posterior root is the “Death Zone” of the knee joint (Figure 1), since any not identifying or unrepaired root tear can accelerate the consequential degenerative cascade of post‐traumatic osteoarthritis in the knee, leading to catastrophic consequences and the need for knee arthroplasty in the later life. The preservation of the meniscus is vital important to the knee; so, let's try to ‘Save the meniscus’ and cure this ‘Silent Epidemic’ on the ‘Death Zone’ of the meniscus in the knee joint.

**Figure 1:**
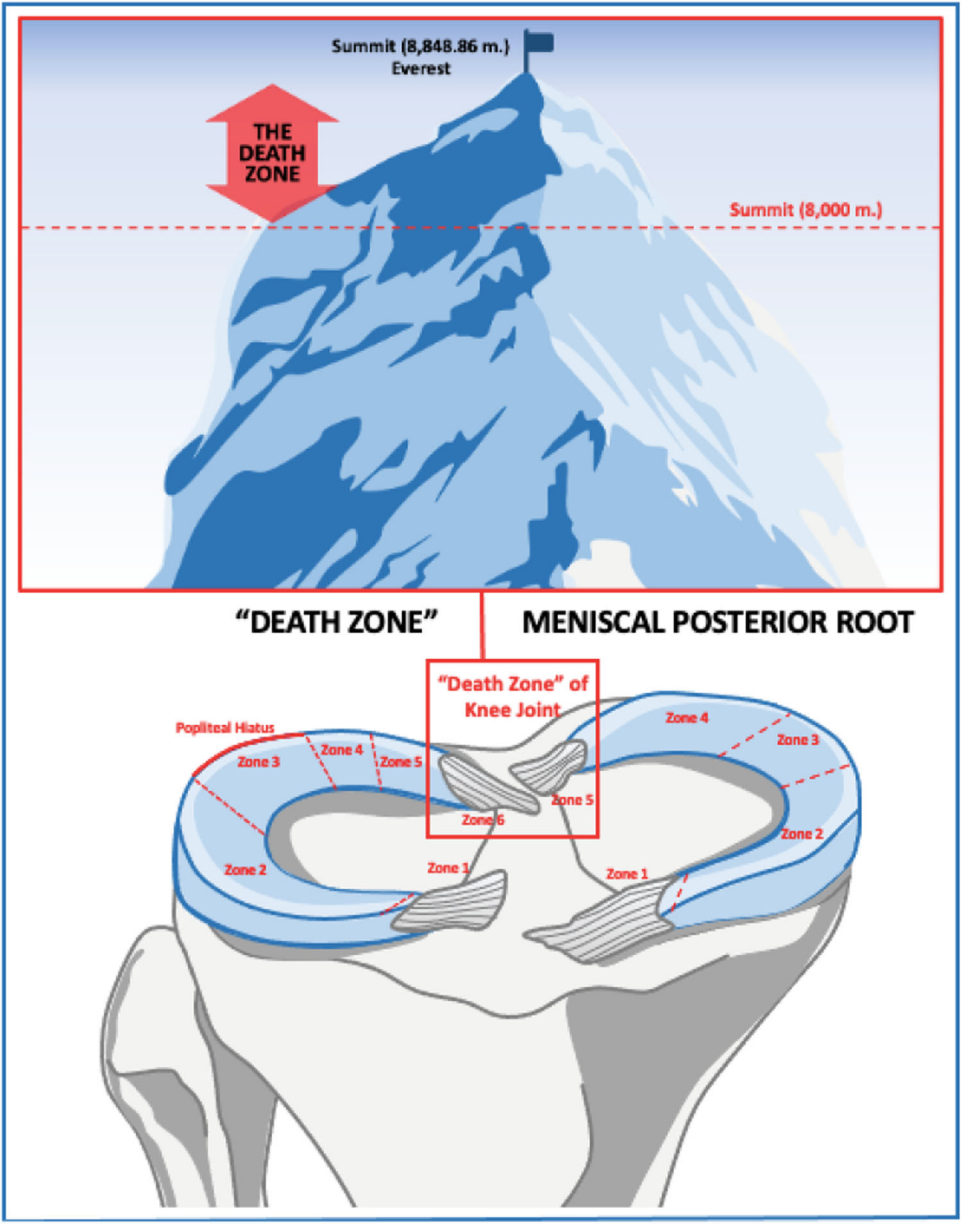
An illustration of the meniscal posterior roots, which represent the ‘death zone’ of the knee joint, like the death zone of 8000 m in the mountains.

## CONFLICT OF INTEREST STATEMENT

The authors declare no conflicts of interest.

## ETHICS STATEMENT

The authors have nothing to report.
